# The Role of Cellular Prion Protein in Cancer Biology: A Potential Therapeutic Target

**DOI:** 10.3389/fonc.2021.742949

**Published:** 2021-09-14

**Authors:** Manqiu Ding, Yongqiang Chen, Yue Lang, Li Cui

**Affiliations:** ^1^Department of Neurology, The First Hospital of Jilin University, Changchun, China; ^2^CancerCare Manitoba Research Institute, CancerCare Manitoba, University of Manitoba, Winnipeg, MB, Canada

**Keywords:** cellular prion protein, cancer, proliferation, metastasis, drug resistance, cancer stem cell

## Abstract

Prion protein has two isoforms including cellular prion protein (PrP^C^) and scrapie prion protein (PrP^Sc^). PrP^Sc^ is the pathological aggregated form of prion protein and it plays an important role in neurodegenerative diseases. PrP^C^ is a glycosylphosphatidylinositol (GPI)-anchored protein that can attach to a membrane. Its expression begins at embryogenesis and reaches the highest level in adulthood. PrP^C^ is expressed in the neurons of the nervous system as well as other peripheral organs. Studies in recent years have disclosed the involvement of PrP^C^ in various aspects of cancer biology. In this review, we provide an overview of the current understanding of the roles of PrP^C^ in proliferation, cell survival, invasion/metastasis, and stem cells of cancer cells, as well as its role as a potential therapeutic target.

## Introduction

**Figure 1:**
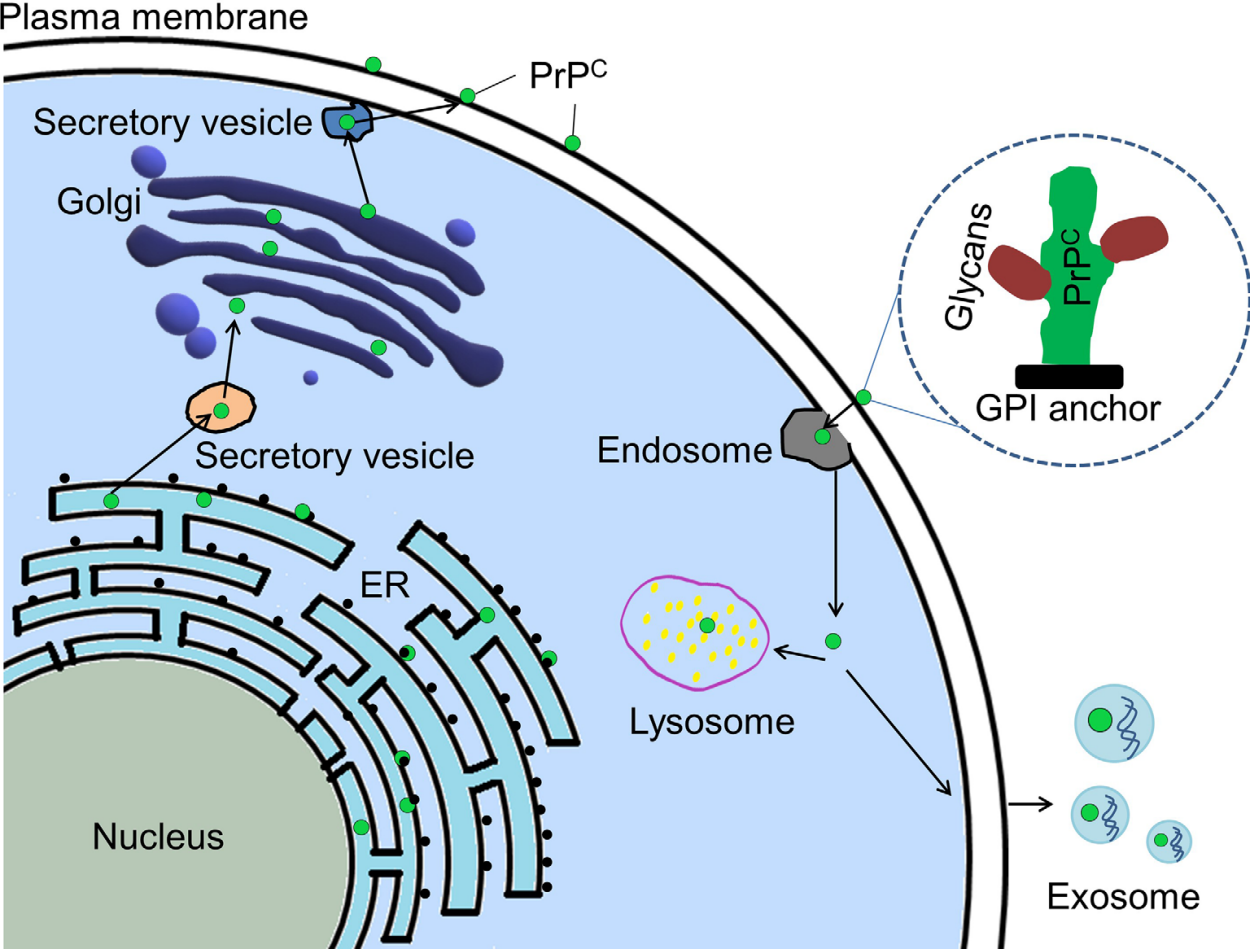
Cellular trafficking pathway of PrP^C^. PrP^C^ (green dot) is synthesized in ribosome attached to ER (endoplasmic reticulum). PrP^C^ is imported to ER where it will be glycosylated and modified by GPI anchor before it is transported into Golgi apparatus for further modification. Mature PrP^C^ is trafficked to plasma membrane and located there by its GPI anchor. Some mature PrP^C^ could be endocytosed for degradation in the lysosome or for being contained in exosomes and secreted outside the cell. PrP^C^, Cellular prion protein; GPI, glycosylphosphatidylinositol.

Prion protein (PrP) is expressed throughout the whole body. It has two isoforms, cellular prion protein (PrP^C^) and its pathogenic form-scrapie prion protein (PrP^Sc^) (1, 2). PrP^Sc^ is well known for its ability to cause a series of neurodegenerative diseases in human and other mammals (1, 3). It results from post-translational conversion of the glycosylphosphatidylinositol (GPI)-anchored PrP^C^ (4, 5). PrP^C^, as a scaffold on the cell surface, recruits different partners to execute its functions being involved in signaling pathways (6). The biosynthetic pathway of PrP^C^ is similar to that of other membrane-attached and secreted proteins (5) (**Figure 1**). It is synthesized in endoplasmic reticulum (ER)-attached ribosomes followed by its import into ER where it is glycosylated and modified by GPI anchor before it is transported into Golgi for further modification. Then PrP^C^ is transported to the cell surface where it can be internalized through endocytic pathway (7). The internalized PrP^C^ can be transported into the lysosome for degradation or be enclosed in exosomes and secreted outside the cells (7). PrP^C^ is mainly attached to lipid rafts on the cell surface *via* its C-terminal GPI anchor (8, 9). It is also located in the cytosol and the nucleus (10–12). Interestingly, PrP^C^ was found in the exosomes secreted by cancer cells (13).

Cancer is the second leading cause of death worldwide. Studies in recent years show that PrP^C^ is involved in various aspects of cancer biology such as cell proliferation, metastasis, cell death, drug resistance and cancer stem cells (14–21). In this review, we summarize the current progress in these aspects.

## PrP^C^ Promotes Cancer Cell Proliferation

PrP^C^ can promote proliferation in cancer cells (22). Liang et al. demonstrated that overexpression of PrP^C^ promoted cell proliferation through activation of the phosphatidylinositide 3-kinase (PI3K) pathway and promotion of the G1/S phase transition by upregulating cyclin D1, in gastric cancer cells (22). PrP^C^ is also involved in G1 to S phase transition in renal adenocarcinoma ACHN and colon adenocarcinoma LS 174T cells (23). Knockdown of PrP^C^ inhibited cell proliferation and amplified the inhibitory effect of fucoidan on cell proliferation by suppressing expression of cyclins and cyclin-dependent kinase (CDK), in HT29 colon cancer cells (24). Interaction of PrP^C^ with the co-chaperone Hsp70/90 organizing protein (HOP) promoted proliferation *via* activating PI3K and extracellular-signal-regulated kinase (ERK1/2) pathways in glioblastomas (GBM) cells (25). Furthermore, HOP-PrP^C^ interaction promoted proliferation of glioblastoma stem-like cells and the decrease expression of PrP^C^ and HOP may work as an effective therapy for GBM in the future (26). Warburg effect refers to the event that cancer cells preferentially use aerobic glycolysis to generate energy and reducing power for their biosynthesis, cell survival and proliferation (27). Overexpression of PrP^C^ mediated Warburg effect by increasing glucose transporter 1 (Glut1) expression which promotes glucose uptake through epigenetic activation of Fyn-HIF-2α-Glut1 pathway in colorectal cancer cells (28). PrP^C^ can also increase cell proliferation by interacting with 37/67 kDa non-integrin laminin receptor (LR/37/67 kDa) and activating downstream ERK1/2 and PI3K/protein kinase B (AKT) signaling pathways in schwannoma cells (29). It promoted proliferation by interacting with Notch1 in pancreatic ductal adenocarcinoma (PDAC) (30). A variant of PrP^C^ with one octapeptide repeat deletion (1-OPRD) is widely present in gastric cancer cell lines and gastric cancer tissues (31). Overexpression of 1-OPRD could promote the proliferation of gastric cancer cells through transcriptional activation of cyclin D3, which facilitated the G1-/S-phase transition in cell cycle (32).

## PrP^C^ Promotes Cancer Cell Invasion/Metastasis

Metastasis leads to more than 90% of cancer-caused death, but its underlying mechanisms still remain poorly understood (33). Christine L et al. divided the process of metastasis into two phases: the first phase is physical translocation of cancer cell from a primary tumor to other distant tissues, and the second phase is colonization of metastatic cancer cells in their new microenvironment (33). EMT refers to epithelial-to-mesenchymal transition (34). Many *in vitro* models show that EMT act as a key process during cancer metastasis (35, 36). Transcription of *Prnp* (the gene encoding PrP) considerably increased during EMT (37). Upregulation of PrP^C^ and dedifferentiation of EMT-like cells were observed in invasive colorectal cancer cells (CRC) (18, 38). Overexpression of PrP^C^ by transfecting pCDNA3.0-*Prnp* in SW480 cells led to EMT whereas, knockdown of *Prnp* in mesenchymal-like LIM2405 cells caused MET (mesenchymal-to-epithelial transition) (18). The mechanisms underlying EMT enhancement by PrP^C^ are largely unclear.

SATB1 (special AT-rich sequence-binding proteins 1) is a nuclear matrix associated protein. It can induce tumor metastasis by altering chromatin structure and upregulating metastasis-associated genes while downregulating tumour-suppressor genes (39, 40). Knockdown of *Prnp* resulted in loss of SATB1 expression and reduction of metastatic capacity in CRC with Fyn and specificity protein 1(SP1) being involved in this process, indicating that PrP^C^ may promote tumor metastasis *via* upregulating the PrP^C^-Fyn-SP1-SATB1 axis (18). PrP^C^ and γ-Syn are overexpressed in CRC (41, 42). They may be involved in colorectal cancer cell metastasis by inducing an endothelial proliferation to differentiation switch (42, 43).

PrP^C^ is highly expressed in metastatic gastric cancer cells and it may promote invasion and metastasis through activation of the mitogen-activated protein kinases (MEK)/ERK pathway and consequent transactivation of matrix metalloproteinase-11(MMP11) (44). MMP11 can promote matrix degradation, inflammation and tissue remodeling (20, 44). Its N-terminal fragment is essential for transducing invasion-promoting signal of PrP^C^ (20, 44). Tissue Inhibitor of Metalloproteinase (TIMP) is endogenous inhibitor for membrane type1-matrix metalloproteinase (MT1-MMP). The binding of TIMP to the GPI anchor of the prion protein generated a membrane-tethered, high-affinity designer TIMP (named “T1^Pr αMT1^” hereafter) which is expressed on the cell surface and co-localized with cellular MTI-MMP (45). Therefore, GPI anchor of PrP^C^ might be used as a potential therapy for renal carcinoma (45).

It was reported that PrP^C^ promoted EMT through the activation of the ERK2/mitogen-activated protein kinase (MAPK1) pathway in colorectal cancer stem cells (46). This is consistent with the notion that the appearance of the CSC (cancer stem cell) phenotype and EMT are intimately connected (19). Notch1 is involved in CSCs (47). It is a downstream effector of PrP^C^ both of which colocalizes on the cell membrane and form an interaction network to promote pancreatic cancer cell metastasis (30). Co-treatment with 5-fluorouracil (5-FU) and melatonin could inhibit colon CSC marker octamer-binding transcription factor 4 (Oct4) *via* downregulation of PrP^C^-Oct4 pathways (48). Tumor-mediated angiogenesis will be suppressed in this process which suggests that cancer metastasis will be inhibited (48). PrP^C^-containing exosomes secreted by CRC could also promote tumor metastasis by increasing the permeability of endothelial cells and the secretion of angiogenic factors (49). This study also demonstrated that the combination of anti-PrP^C^ and 5-FU downregulated tumor progression (49).

The immune system is one of the key pathways to control cancer development and metastasis. Regulatory T cells (Tregs), which have immunosuppressive activity (50), are one of the main targets of cancer immunotherapy (51). By constructing a lung metastatic model of melanoma in Prnp0/0 and Tga20 mice, it was demonstrated that the increased expression of PrP^C^ induces the development of Tregs by upregulating transforming growth factor-beta (TGF-β) and programmed death ligand-1(PD-L1), thereby promoting tumor progression (52).

**Figure 2:**
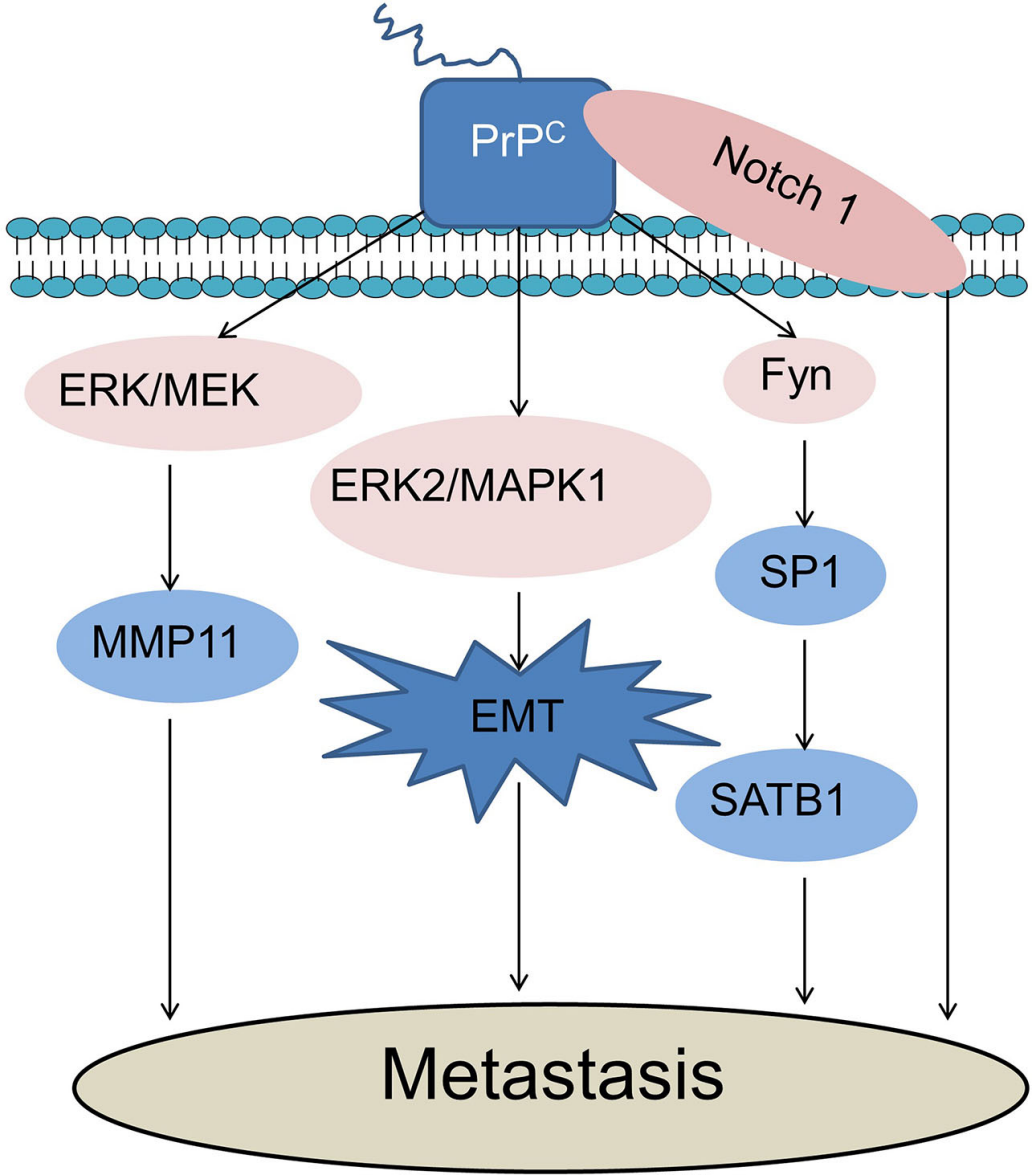
PrP^C^ promotes cancer cell metastasis. PrP^C^ could promote cancer cell metastasis through activation of the MEK/ERK pathway and consequent transactivation of MMP11. PrP^C^ promotes EMT through the activation of the ERK2/MAPK1 pathway during cancer metastasis. PrP^C^ could promote tumor metastasis via up-regulating the PrP^C^-Fyn-SP1-SATB1 axis. Notch1 and PrP^C^ could form an interaction network to promote cancer cell metastasis. ERK, Extracellular-signal-regulated kinase; MEK, Mitogen-activated protein kinases; MMP11, Matrix metalloproteinase-11; MAPK, Mitogen-activated protein kinase; EMT, Epithelial-mesenchymal transition; SATB1, Special AT-rich sequence-binding proteins 1; SP1, Specificity protein 1.

Many studies have demonstrated that PrP^C^ expression promotes cancer cell metastasis (**Figure 2**). However, one study showed that knockout of *Prnp* (*Prnp* ^0/0^) in mesenchymal embryonic mouse cells transformed by Ras/Myc led to more incidence of lung metastasis due to increased expression of α_V_β_3_-integrin (53). This suggest that more studies are required to clarify the roles of PrP^C^ in cancer metastasis.

## PrP^C^ Promotes Cancer Cell Drug Resistance

One major challenge for cancer treatment is drug resistance. Various mechanisms can contribute to cancer drug resistance (54). The most studied mechanisms involving the roles of PrP^C^ in cancer drug resistance include multi-drug resistance (MDR) and inhibition of cell death. Multi-drug resistance (MDR) refers to the ability of cancer cells to survive against a wide range of anti-cancer drugs (55). Cell death can be classified into three main types including apoptosis (Type I programmed cell death), autophagic cell death (Type II programmed cell death) and necrosis (56). Apoptosis is characterized by cell shrinkage, membrane blebbing, chromatin condensation, DNA fragmentation and caspase activation. Autophagic cell death is induced by the over-activation of autophagy that is an intracellular lysosomal degradation process. Necrosis is a non-programmed cell death. It is caused by sudden results to the cells and is characterized by breakage of plasma membrane followed by cytoplasmic leakage.

Upregulation of PrP^C^ can lead to drug resistance in different types of cancers cells (57–59). In colorectal cancer cells, PrP^C^ is involved in 5-FU resistance by increasing cell survival and proliferation *via* activating PI3K-Akt signaling pathway and the expression of cell cycle-associated proteins (59). PrP^C^ overexpression led to resistance of colorectal cancer LS174T cells to doxorubicin-induced apoptosis by upregulation of the inhibitors of apoptosis proteins (IAPs) (60). Upregulation of PrP^C^ leads to increased superoxide dismutase and catalase activities and decreased endoplasmic reticulum stress and apoptosis, which results in oxaliplatin resistance in colorectal cancer cells (61, 62). In gastric cancer cells, PrP^C^ can promote drug resistance by different mechanisms. PrP^C^ coexists with MGr1-Antigen/37 kDa laminin receptor precursor (MGr1-Ag/37LRP) to promote MDR in gastric cancer cells by inhibiting apoptosis *via* activation of the PI3K/AKT signaling pathway (63). Octarepeat peptides of PrP may be involved in gastric cancer MDR by increasing the activities of antioxidant enzymes (64). PrP^C^ can promote MDR by upregulating the multidrug resistance protein (P-gp) and suppressing apoptosis in gastric and breast cancer cells (65, 66). Overexpression of PrP^C^ promotes resistance to TNF-α-induced apoptosis by inhibiting Bcl-2-associated X protein (Bax) expression in renal adenocarcinoma ACHN cells (23).

PrP^C^ can be found on the cell surface by attaching to the cell membrane and outside the cells being contained in exosomes which are secreted from the cells (67, 68). The secreted PrP^C^ in tumor microenvironment binds to doxorubicin to prevent it from entering the nucleus and intercalating into DNA to induce cell death; and breast cancer patients with high levels of serum PrP^C^ are at high risk of relapse following doxorubicin treatment (13). PrP synthetic peptide (amino acid residues 105 - 120 of the human prion protein) can protect schwannoma cells from H_2_O_2_-mediated cell death (29).

PrP^C^ has been shown to protect cancer cells from apoptosis and autophagic cell death (69). PrP^C^ inhibits apoptosis in neurons and in cancer cells (70). PrP^C^ upregulation inhibits apoptosis induced by Bax expression, serum starvation and anti-cancer drug treatments (57, 70, 71). PrP^C^ can bind to the C-terminus of the anti-apoptotic protein Bcl-2 to form a dimer inhibiting apoptosis (72). When PrP^C^ is upregulated, Bcl-2/Bax ratio increases, resulting in anti-apoptosis in breast carcinoma MCF-7 cells (71). Tumor necrosis factor-related apoptosis-inducing ligand (TRAIL) is a ligand for death receptors which can induce cancer cell apoptosis (73). Downregulation of PrP^C^ sensitizes adriamycin-resistant human breast cancer cells to TRAIL-induced apoptosis by increasing Bax/Bcl-2 ratio (58). PrP^C^ inhibited TRAIL-induced apoptosis under hypoxia in human colon carcinoma cells (74). Akt was activated by PrP^C^ to prevent TRAIL-induced apoptosis (75, 76). PrP^C^ also activated PI3K/Akt signaling pathway contributing to its anti-Bax function by preventing the pro-apoptotic conformational changes of Bax at the early step of Bax activation (71). Moreover, PrP^C^ protected lung and pancreatic cancer cells from apoptosis through downregulation of unfolded protein response (UPR) (77).

Autophagy is an evolutionarily conserved catabolic process in eukaryotic cells, in which unnecessary or dysfunctional cytosolic components are degraded and recycled through lysosomes (78). During autophagy (macroautophagy), cytosolic components (cargos) are surrounded by a phagophore which will expands and encloses to form the characteristic double-membraned structure autophagosome. Then, autophagosome will fuse with the lysosome to form autolysosome where cargos are degraded to generate small molecules that can be used for biosynthesis and energy production for cell survival, under stress conditions such as starvation (79). However, when autophagy is over-enhanced, it can induce cell death (autophagic cell death/autophagy-induced cell death) (79). Barbieri et al. demonstrated for the first time that PrP^C^ can modulate autophagic cell death in glial tumor cells (80). They demonstrated that PrP^C^ silencing resulted in inhibition of Mammalian target of rapamycin (mTOR) kinase activity in T98G glioma cells, promoting autophagy leading to autophagic cell death (80). Furthermore, PrP^C^ inhibited autophagy by activating the antioxidant enzyme SOD (81). Since autophagy is mainly a pro-cell survival mechanism, it is expected that PrP^C^ may antagonize drug resistance by inhibiting autophagy in cancer cells.

**Figure 3:**
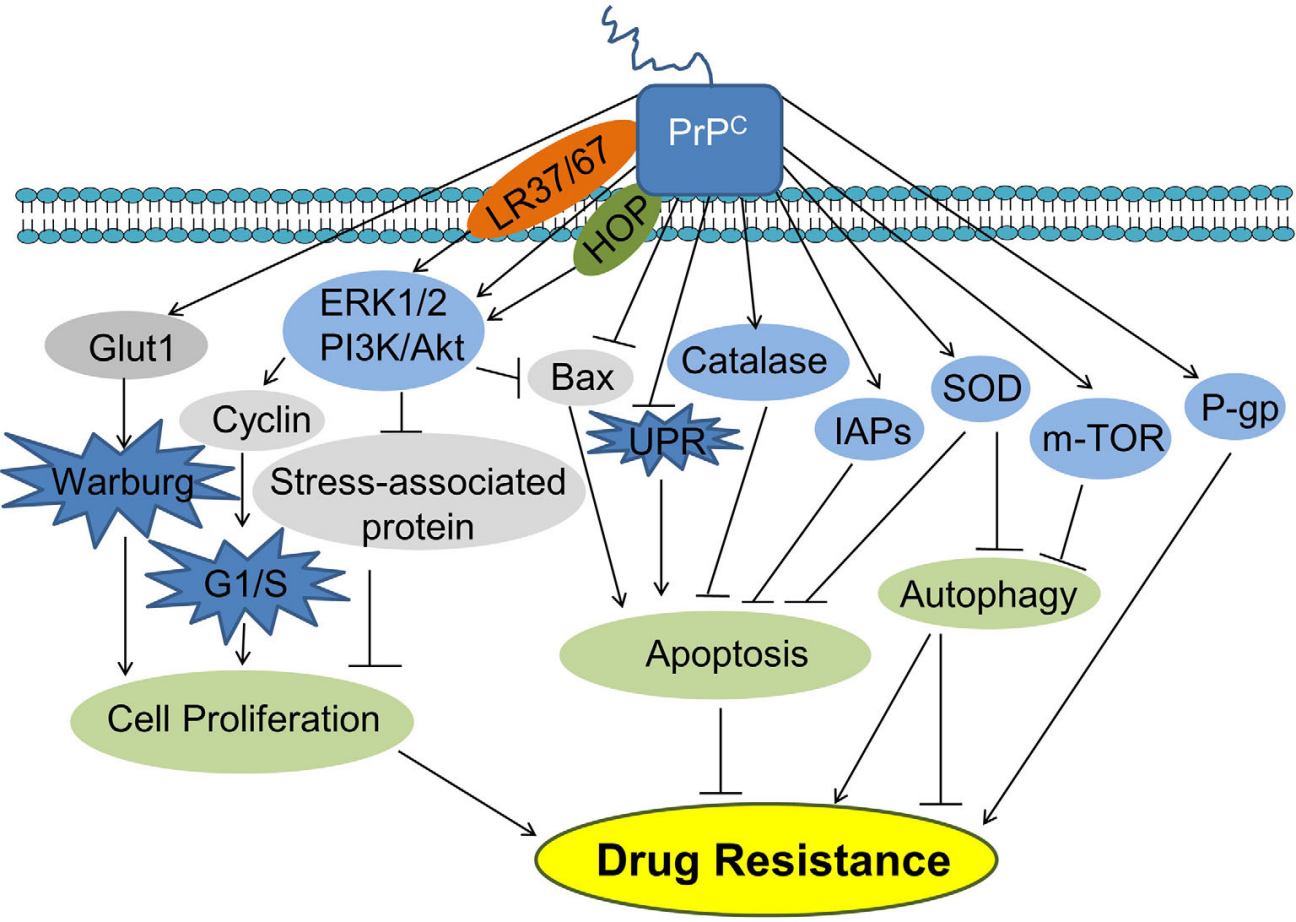
PrP^C^ promotes cancer cell drug resistance. PrP^C^ can promote cancer cell drug resistance by promoting cell proliferation and inhibiting apoptosis. PrP^C^ can also suppress autophagy inhibiting or promoting drug resistance. HOP, Hsp70/90 organizing protein; IAPs, Inhibitors of apoptosis proteins; Glut1, Glucose transporter 1; PI3K, Phosphatidylinositide 3-kinase; AKT, Protein kinase B; Bax, Bcl-2-associated X protein; UPR, Unfolded protein response; SOD, Superoxide dismutase; P-gp, P-glycoprotein.

One study showed that tumor resistance to radiotherapy was also associated with the increased PrP^C^ (82). In neuroblastoma, breast, and colorectal cancer cell lines, ionizing radiation (IR) can increase the expression of PrP^C^ by activating ATM-TAK1-PrP^C^ pathway, thereby leading to the resistance to radiotherapy of tumor cells (82). Taken together, PrP^C^ can modulate various signaling pathways contributing to cancer drug resistance (**Figure 3**).

Although the overexpression of PrP^C^ in cancer cells results in therapy-resistance, researchers have taken advantage of this characteristic to synthesize PrP^C^-Apt-functionalized doxorubicin-oligomer-AuNPs (PrP^C^-AptDOa) which could target PrP^C^-overexpressed CRC (83). PrP^C^-AptDOa inhibited CRCs proliferation and induced apoptosis more significantly than free Dox at the cellular level (83). However, PrP^C^ is also expressed in normal cells, such as neurons and neuroglia. Therefore, the challenge for cancer treatment is to specifically target PrP^C^ in cancer cells. In addition, further studies of PrP^C^-AptDOa should be conducted in an animal model and clinical trials to clarify its therapeutic effects and side effects on individuals.

## PrP^C^ Promotes Cancer Stem Cell Development

Cancer stem cells (CSCs) are a small subpopulation of cancer cells with the capacities of self-renewal, differentiation and tumorigenicity (84). PrP^C^ is engaged in different types of stem cells, such as hematopoietic stem cells (HSCs), gland stem cells, bone marrow-derived human mesenchymal stem cells (MSCs) and human embryonic stem(ES) cells (85–88). Studies have indicated that PrP^C^ is also involved in CSCs. PrP^C^ protected Oct4, a marker of colon cancer stem cells, from degradation by inducing heat shock protein 1 like (HSPA1L) when in response to co‐treatment with 5‐FU and melatonin (48). One study indicated that PrP^C^ was highly expressed in consensus molecular subgroup (CMS4), a subtype of CRC with higher malignancy, and affected the prognosis of CRC as an upstream molecule in the PrP^C^-ILK-IDO1 axis (89). PrP^C^ promoted EMT of colorectal cancer stem cells *via* activation of the ERK2 (MAPK1) pathway to increase cell metastasis (46). CD44 is a CSC marker and critical regulator of cancer stemness (90). PrP^C^ is co-expressed with CD44 in colorectal CSCs (46). PrP^C^ and Hsp70/90 organizing protein (HOP) acted together to regulate self-renewal, proliferation and migration in glioblastoma (GBM) stem-like cells (26). Downregulation of PrP^C^ decreased stem cell-like properties of human GBM CSCs (91). Downregulation of PrP^C^ in models of prion disease through immune, genetic and other mechanisms has achieved some progress. Application of anti-PrP antibodies have been proposed as a promising treatment many decades ago (92, 93). A recent study reported that transgenic mice expressing elk PrP (TgElk) benefited from active PrP vaccination (94). Minikel et al. demonstrated that PrP-lowering antisense oligonucleotides (ASOs) worked *via* an RNAase-H dependent mechanism and has certain therapeutic effect on prion-infected mice (95). Minikel et al. also proposed that loss-of-function variant of *Prnp* could be potential targets for prion disease inhibitory drugs (96). The application of these PrP^C^-lowering approaches may provide novel cancer therapies by targeting CSCs.

## Conclusion

Prion protein (PrP) is expressed in nervous system and other organs (97). There are two forms of PrP, including normal PrP^C^ and disease causing PrP^Sc^. PrP^C^ misfolding and aggregation can cause fatal neurodegenerative conditions (98). Studies in recent years show that it also plays a role in cancer. PrP^C^ can stimulate cancer progression by promoting cancer cell proliferation, invasion/metastasis, drug resistance, and cancer stem cell development. Therefore, targeting PrP^C^ is a novel approach for cancer treatment.

## Author Contributions

MD and YC conceived the topic and designed the outline of this review. MD contributed to the manuscript writing and prepared the figures and tables. YC modified the language. LC, YC and YL critically revised the manuscript. All authors contributed to the article and approved the submitted version.

## Funding

This work was supported by a grant from the National Natural Science Foundation of China (82071351).

## Conflict of Interest

The authors declare that the research was conducted in the absence of any commercial or financial relationships that could be construed as a potential conflict of interest.

## Publisher’s Note

All claims expressed in this article are solely those of the authors and do not necessarily represent those of their affiliated organizations, or those of the publisher, the editors and the reviewers. Any product that may be evaluated in this article, or claim that may be made by its manufacturer, is not guaranteed or endorsed by the publisher.

## Abbreviations

Bax, Bcl-2-associated X protein; Bcl, B cell leukemia oncogene; CRC, Colorectal cancer cell; CSC, Cancer stem cell; EMT, Epithelial-Mesenchymal Transition; ERK, Extracellular-signal-regulated kinase; 5-FU, 5-fluorouracil; GBM, Glioblastomas; GPI, Glycosylphosphatidylinositol; HOP, Hsp70/90 organizing protein; HSCs, Hematopoietic stem cells; MDR, Multi-drug resistance; MET, Mesenchymal-to-epithelial transition; MMP11, Matrix metalloproteinase-11; MSCs, Mesenchymal stem cells; Oct4, Octamer-binding transcription factor 4; 1-OPRD, One octapeptide repeat deletion; PDAC, Pancreatic ductal adenocarcinoma; P-gp, P-glycoprotein; PI3K, Phosphatidylinositol 3 kinase; PrP^C^, Cellular prion protein; PrP^Sc^, Scrapie prion protein; PrP^C^-AptDOa, PrP^C^-Apt-functionalized doxorubicin-oligomer-AuNPs; SATB1, Special AT-rich sequence-binding proteins 1; SOD, Superoxide dismutase; TIMP, Tissue Inhibitor of Metalloproteinase; TNF-α, Tumor Necrosis Factor-α; TRAIL, Tumor necrosis factor-related apoptosis-inducing ligand; UPR, Unfolded protein response.
